# CHALLENGES IN IDENTIFYING PERSONS WITH NEURODEGENERATIVE DISEASES USING HEALTH ADMINISTRATIVE DATABASES

**DOI:** 10.1093/geroni/igz038.058

**Published:** 2019-11-08

**Authors:** Laura C Maclagan, Serena Soleimani, Agessandro Abrahao, Lorne Zinman, Michael A Campitelli, Liisa Jaakkimainen, Colleen J Maxwell, Susan E Bronskill

**Affiliations:** 1 Institute for Clinical Evaluative Sciences (ICES), Toronto, Ontario, Canada; 2 Institute of Medical Science, University of Toronto, Toronto, Ontario, Canada; 3 Sunnybrook Health Sciences Centre, Toronto, Ontario, Canada; 4 ICES, Toronto, Ontario, Canada; 5 University of Waterloo, Waterloo, Ontario, Canada

## Abstract

Health administrative databases can be used to quantify prevalence and incidence of neurodegenerative diseases and their impact on health service utilization outcomes at the population level. Algorithms based on diagnosis codes and health service patterns can be used to identify persons suspected to have a neurodegenerative disease. Previous studies have developed and validated algorithms to identify persons with Alzheimer’s and related dementias using primary care medical records as the reference standard, however, little previous work has focused on developing algorithms for rare neurodegenerative diseases including amyotrophic lateral sclerosis (ALS). This session will discuss challenges in developing algorithms to identify persons with neurodegenerative diseases accurately and opportunities to improve existing definitions using novel data sources including electronic medical record databases. Preliminary findings regarding the development of an ALS algorithm will be presented.

